# A dual-mode fiber-shaped flexible capacitive strain sensor fabricated by direct ink writing technology for wearable and implantable health monitoring applications

**DOI:** 10.1038/s41378-023-00634-9

**Published:** 2023-12-21

**Authors:** Chi Zhang, Wenyu Ouyang, Lei Zhang, Dachao Li

**Affiliations:** State Key Laboratory of Precision Measuring Technology and Instruments, Tianjin University, 300072 Tianjin, China

**Keywords:** Nanoscience and technology, Materials science

## Abstract

Flexible fiber-shaped strain sensors show tremendous potential in wearable health monitoring and human‒machine interactions due to their compatibility with everyday clothing. However, the conductive and sensitive materials generated by traditional manufacturing methods to fabricate fiber-shaped strain sensors, including sequential coating and solution extrusion, exhibit limited stretchability, resulting in a limited stretch range and potential interface delamination. To address this issue, we fabricate a fiber-shaped flexible capacitive strain sensor (FSFCSS) by direct ink writing technology. Through this technology, we print parallel helical Ag electrodes on the surface of TPU tube fibers and encapsulate them with a high dielectric material BTO@Ecoflex, endowing FSFCSS with excellent dual-mode sensing performance. The FSFCSS can sense dual-model strain, namely, axial tensile strain and radial expansion strain. For axial tensile strain sensing, FSFCSS exhibits a wide detection range of 178%, a significant sensitivity of 0.924, a low detection limit of 0.6%, a low hysteresis coefficient of 1.44%, and outstanding mechanical stability. For radial expansion strain sensing, FSFCSS demonstrates a sensitivity of 0.00086 mmHg^−1^ and exhibits excellent responsiveness to static and dynamic expansion strain. Furthermore, FSFCSS was combined with a portable data acquisition circuit board for the acquisition of physiological signals and human‒machine interaction in a wearable wireless sensing system. To measure blood pressure and heart rate, FSFCSS was combined with a printed RF coil in series to fabricate a wireless hemodynamic sensor. This work enables simultaneous application in wearable and implantable health monitoring, thereby advancing the development of smart textiles.

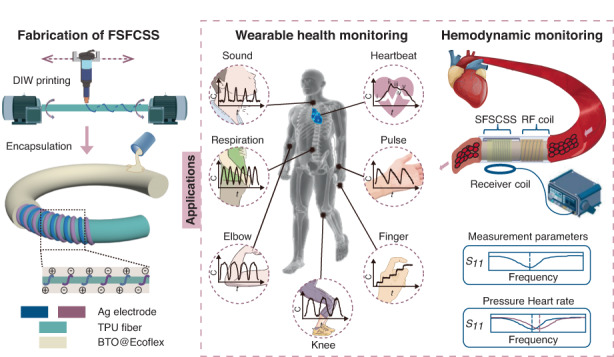

## Introduction

With the emergence of emerging technologies, including flexible electronics, wearable devices, and foldable devices, fiber-shaped strain sensors have attracted widespread attention as compact, easily integrated, portable, and highly comfortable wearable electronics^1^. Various fiber-shaped strain sensors have been developed and widely applied in various fields, including medical health monitoring, sports tracking, smart wearable devices, human‒machine interaction, virtual reality, robotics, and more^2–5^. Fiber-shaped strain sensors can be divided into resistive^6^, piezoelectric^7^, capacitive^8^, triboelectric^9^, and magnetoelastic^10–12^ types based on their sensing mechanisms. Generally, piezoelectric and triboelectric strain sensors lack static testing capabilities and are sensitive to external vibrations and shocks, leading to significant measurement interference^13^. Resistive strain sensors are sensitive to the temperature of the working environment and exhibit nonlinear output characteristics, while significant hysteresis phenomena lead to signal drift^14^. Sensors based on the magnetoelastic effect are highly sensitive to dynamic pressure sensing but cannot perform static pressure sensing^15–17^. As a comparison, fiber-shaped capacitive strain sensors show potential candidate features and significant advantages, such as high sensitivity, low-temperature effects, low power consumption, and dynamic and static response capabilities. Therefore, these sensors are more suitable for long-term and continuous human health monitoring applications.

Currently, the traditional manufacturing methods for fiber-shaped strain sensors typically involve sequential coating and solution extrusion methods^18^. Depending on the desired structure of the fiber-shaped electronics, sequential coating involves immersing the substrate in one or more liquid coatings to form the strain-sensitive layers in sequence. For example, Lee et al.^8^ fabricated a hollow capacitive strain sensor composed of two conductive fibers by coating and measured small tensile strains occurring on connective tissues. Zhang et al.^19^ proposed a single fiber strain sensor based on graphite flakes and silk fibers by a dry-Meyer-rod-coating process and successfully integrated sensors into a multidirectional strain sensor for monitoring multiaxial strain. Fiber-shaped strain sensors fabricated using sequential coating methods are often limited by the poor stretchability of conductive and sensitive materials. This limitation results in a limited overall stretching range for the sensors. Additionally, the multiple interfaces involved in the layer-by-layer coating process are prone to detachment when the sensors are repeatedly stretched. This leads to decreased stability and larger testing errors for the sensors. Similarly, solution extrusion usually requires the extrusion of precursor solutions that contain various functional materials through a nozzle to produce precise flow, which then solidifies into long functional fibers. Zheng et al.^20^ fabricated a highly stretchable CNT@Ecoflex strain sensor through an integrated coaxial wet spinning process, which is used to accurately detect subtle and large-scale human movements. However, the solution extrusion method is a destructive processing method that results in poor stretchability of fiber electronic devices because the materials within the mixed solution jointly determine their electromechanical performance. Additionally, this method requires the use of potentially toxic organic solvents, which compromises the safety and biocompatibility of wearable electronics. Notably, some implantable health monitoring applications urgently require fiber-shaped electronics with customizable size and shape, as well as adjustable sensitivity and sensing range. Therefore, a customizable and directly printed fiber electronic processing method is needed to address the aforementioned challenges.

Direct ink writing (DIW) technology is an additive manufacturing process that directly prints sensitive materials onto a substrate using a nozzle. It offers several advantages, such as pattern customization, diverse material options, and low cost^21^. Additionally, the DIW method does not destroy the substrate material, allowing for the utilization of its excellent mechanical properties, avoiding interface detachment, and enhancing biocompatibility. Through DIW, stretchable electrode structures, such as snake-shaped, horseshoe-shaped, and 3D helical structures, can be easily fabricated, further expanding the detection range of fiber-shaped strain sensors. These benefits present a potential solution to the aforementioned challenges.

Here, a fiber-shaped flexible capacitive strain sensor (FSFCSS) based on DIW technology is developed. The parallel helical Ag electrode is custom printed on the stretchable TPU tube fiber surface, with BTO@Ecoflex serving as the dielectric and encapsulation layer, endowing FSFCSS with excellent tensile strain and expansion strain sensing performance. Therefore, the FSFCSS can sense dual-model strain, namely, axial tensile strain and radial expansion strain. For axial tensile strain sensing, FSFCSS exhibits a wide detection range (178%), excellent sensitivity (0.924), low detection limit (0.6%), and extremely low hysteresis coefficient (1.44%). For radial expansion strain sensing, FSFCSS exhibits a sensitivity of 0.00086 mmHg^−1^ and demonstrates excellent responsiveness to static and dynamic expansion pressures. Based on the excellent performance of the dual-mode FSFCSS, we demonstrate applications in the field of physiological signal monitoring and human‒machine interactive smart gloves. In addition, a conceptual implantable wireless blood hemodynamic sensor is demonstrated.

## Results and discussion

### Fabrication and characterization of the FSFCSS

**Fig. 1:**
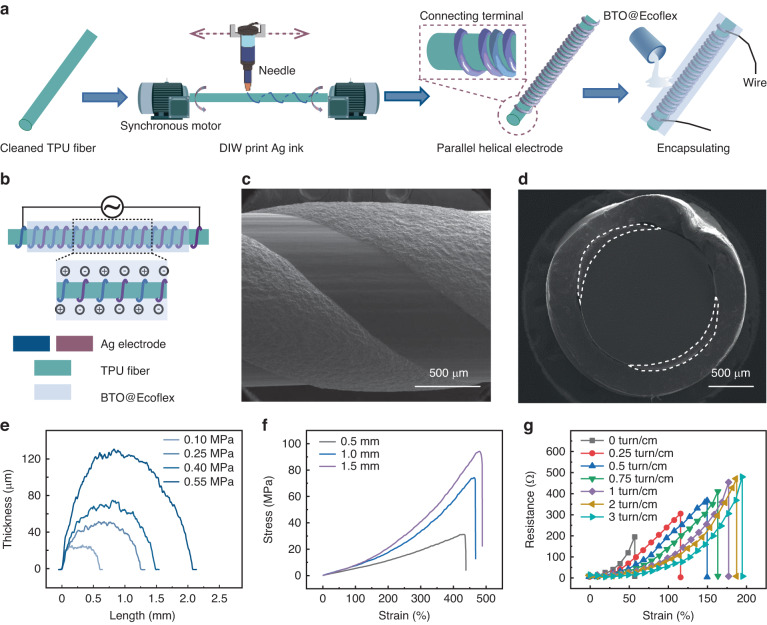
**Fabrication and characterization of the FSFCSS**. **a**, **b** The processing flowchart and structural schematic diagram of FSFCSS. **c**, **d** The surface and cross-sectional SEM images of the Ag electrode printed on the elastic TPU fiber. **e** The width and thickness of the Ag electrode printed with different air pressures. **f** Stress‒strain curves of TPU fibers printed with a 1 turn/cm helical Ag electrode. **g** The tensile resistance of Ag electrodes with different turn densities after encapsulation by BTO@Ecoflex

As shown in Fig. 1a, b, FSFCSS presents a three-layer structure, namely, the elastic TPU fiber, parallel helical Ag electrodes, and BTO@Ecoflex encapsulation layer. The TPU fiber was selected as the substrate material due to its excellent elasticity (over 400%) and high rebound rate (>95%). A parallel helical structure can convert linear strain into bending strain uniformly, greatly enhancing the strain range of printed Ag ink electrodes. BTO@Ecoflex is used as the encapsulation layer and dielectric layer due to its high dielectric properties and good biocompatibility. The preparation process was performed as follows: the TPU fiber was first treated by plasma and surfactant to achieve a hydrophilic surface, a JTO fixture fixed on a synchronous motor was used to fix and provide rotational motion to the TPU fibers, a pneumatic nozzle printed Ag ink onto the surface of TPU fiber with a linear motion, and the ink extrusion speed of the nozzle was independently controlled by a pneumatic valve.

The surface and cross-sectional SEM images of the printed FSFCSSs are displayed in Fig. 1c, d. The Ag ink exhibits good adhesion to the TPU fiber and presents a flat and defect-free electrode surface with smooth and continuous edges and without ink diffusion. The interface between the Ag ink and TPU fibers is flat, uniform, tightly integrated, and without noticeable gaps, and the encapsulation layer of BTO@Ecoflex is uniform, as shown in Supplementary Fig. S1. The BTO@Ecoflex dielectric layer tightly wraps the printed Ag electrode, which significantly enhances the robustness of the Ag electrode. Moreover, the printed linewidth and helical structural parameters, such as length, parallel spacing, and turn density, of the Ag electrode can be directly determined by DIW printing equipment. As shown in Fig. 1e, as the printing pressure increases, the volume of extruded Ag ink per unit of time also increases, resulting in wider and thicker Ag electrodes on the surface of the TPU fibers.

The mechanical and electrical conductive properties of TPU fibers printed with helical Ag electrodes were further studied. Figure 1f and Supplementary Fig. S2 exhibit the stress‒strain curves of TPU fibers with 0.5, 1.0, and 1.5 mm diameters with and without printed Ag electrodes. The results reveal that TPU fibers themselves exhibit excellent tensile properties of >400% strain, and the printed Ag electrodes have a minimal effect on the tensile and mechanical properties of TPU fibers. Figure 1g and Supplementary Fig. S3 present the resistance of encapsulated and nonencapsulated helical Ag ink electrodes with different helical turn densities under different strains. The nonencapsulated Ag ink used in this study exhibits an inherent stretchability of 35%. As the turn density increases, the strain sensitivity of the resistance of the Ag electrode gradually decreases, which demonstrates that the helical structure can serve as a buffer under tension strain. With increasing helical turn density to 3 turns/cm, the breakage point strain increases to 186%, which also supports the previous results. Furthermore, the robust encapsulation of BTO@Ecoflex enhances the strong adhesion between the Ag ink electrode and the TPU fiber interface, acting as a protective barrier for the printed helical Ag ink electrode and further improving the stretchability of FSFCSS.

Overall, the FSFCSS fabricated by DIW printing technology exhibits excellent tensile and conductivity properties. Along with the fibrous structure and the ability to customize its structural parameters, the SFSCSS is suitable as a capacitive strain sensor for wearable and implantable applications.

### Working mechanism of FSFCSS

FSFCSS, as a capacitive sensor, can have two working modes, namely, the axial tensile strain sensing mode and radial expansion strain sensing mode. As is well known, the capacitance can be defined by the formula^22^:

1 $$C=\frac{\epsilon S}{d}$$

where *C* is the capacitance of FSFCSS, *ϵ* is the dielectric constant of BTO@Ecoflex, and *S* and *d* are the area and distance between electrodes, respectively.

**Fig. 2:**
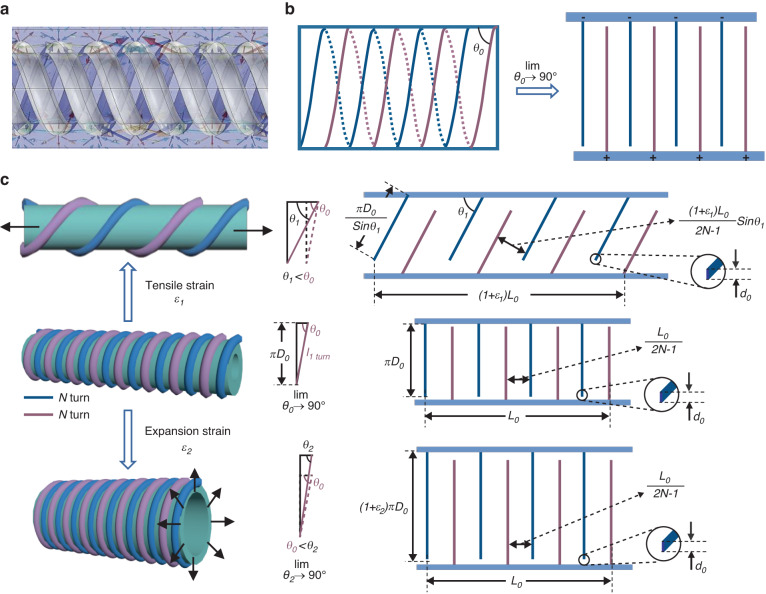
**Working mechanism of FSFCSS**. **a** COMSOL simulation result of the electric field finite element simulation of the FSFCSS. **b** Schematic diagram of the process of converting a 3D helical electrode into a 2D interdigital electrode. **c** The geometrical representation of the FSFCSS of the axial tensile strain sensing mode and radial expansion strain sensing mode

Considering the small distance between the parallel helical Ag electrodes and the alternating structure, as shown in Fig. 2a, b, the 3D parallel helical electrode structure can be equivalently modeled as a 2D interdigital electrode with equal parameters, as previously reported^23^. Therefore, the *N*-turn helical Ag ink electrode can be equivalent to (2*N*−1) interdigital electrodes. Because the thickness of the printed electrode is much smaller than the fiber diameter, the change in electrode thickness during the straining process is ignored. In addition, the angle between the trajectory of the helical electrode and the TPU fiber axis is *θ*_*0*_. Therefore, the geometric relationship of the length of a single-turn helical electrode *l*_*1 turn*_ can be defined as follows:

2 $${l}_{1{turn}}=\frac{\pi {D}_{0}}{\sin {\theta }_{0}}$$

When the turn density is large enough, the angle *θ*_*0*_ approaches 90° (${\theta }_{0}\to 90^\circ$, $\sin {\theta }_{0}\to 1$), so the length of a single-turn helical electrode can be approximated as the perimeter of the TPU fiber substrate. Thus, the *C*_*0*_ of FSFCSS can be further calculated as:

3 $${C}_{0}=\frac{\left(2N-1\right){\epsilon }_{0}{d}_{0}\pi {D}_{0}}{\frac{{L}_{0}}{2N-1}}=\frac{{\left(2N-1\right)}^{2}{\epsilon }_{0}{d}_{0}\pi {D}_{0}}{{L}_{0}}$$

where *N* is the number of turns of one helical electrode, *ϵ*_*0*_ is the dielectric constant of BTO@Ecoflex, *d*_*0*_ is the electrode thickness, *D*_*0*_ is the diameter of the TPU fiber, and *L*_*0*_ is the total length of the FSFCSS.

For the axial tensile strain and radial expansion strain sensing modes, as shown in Fig. 2c, FSFCSS is assigned a tensile strain *ε*_*1*_ and an expansion strain *ε*_*2*_, respectively. In this case, the angle between the trajectory of the helical electrode and the TPU fiber axis is defined as *θ*_*1*_ (${\theta }_{1} < {\theta }_{0}$) and *θ*_*2*_ (${\theta }_{0} < {\theta }_{2}\to 90^\circ$, $\sin {\theta }_{2}\to 1$), and the capacitances *C*_*Tension*_ and *C*_*Expansion*_ can be defined as^24^:

4 $${C}_{{Tension}}=\frac{\left(2N-1\right){\epsilon }_{0}{d}_{0}\frac{\pi {D}_{0}}{\sin {\theta }_{1}}}{\frac{\left(1+{\varepsilon }_{1}\right){L}_{0}}{2N-1}\sin {\theta }_{1}}=\frac{{C}_{0}}{(1+{\varepsilon }_{1}){\sin }^{2}{\theta }_{1}}$$

5 $${C}_{{Expansion}}=\frac{\left(2N-1\right){\epsilon }_{0}{d}_{0}\pi {\left(1+{\varepsilon }_{2}\right)D}_{0}}{\frac{{L}_{0}}{2N-1}}=\left(1+{\varepsilon }_{2}\right){C}_{0}$$

where

6 $$\sin {\theta }_{1}=\sin \left({\tan }^{-1}\frac{\pi {D}_{0}}{\frac{\left(1+{\varepsilon }_{1}\right){L}_{0}}{2N-1}}\right)$$

Sensitivity is a key indicator used to evaluate the performance of sensors, and the gauge factor (GF) is used to indicate the sensitivity of the capacitive strain sensor, which can be defined as ${GF}=\frac{\triangle C}{{C}_{0}\varepsilon }$. A higher sensitivity indicates that the sensor is more sensitive to small strains and can measure strains more accurately. For the above two sensing modes, the sensitivity *GF*_*Tension*_ and *GF*_*Expansion*_ can be defined as follows:

7 $${{GF}}_{{Tension}}=\frac{{C}_{{Tension}}-{C}_{0}}{{C}_{0}{\varepsilon }_{1}}=\frac{1}{{\varepsilon }_{1}(1+{\varepsilon }_{1})}\,\cdot\, \frac{1}{{\sin }^{2}{\theta }_{1}}-\frac{1}{{\varepsilon }_{1}}$$

8 $${{GF}}_{{Expansion}}=\frac{{C}_{{Expansion}}-{C}_{0}}{{C}_{0}{\varepsilon }_{2}}=1$$

From Equation 7, it can be seen that under the same strain, $\frac{1}{{\varepsilon }_{1}(1+{\varepsilon }_{1})}$ and $\frac{1}{{\varepsilon }_{1}}$ are constants, so the *GF*_*Tension*_ of the axial tensile strain sensing mode is only related to the angle *θ*_*1*_, which is further determined by the customizable total length *L*_*0*_ and total turns *N* of the printed sensor. The *GF*_*Expansion*_ of the expansion strain sensing mode is an inherent characteristic of the sensor under ideal expansion.

### Electrical output characteristics of dual-mode FSFCSS

**Fig. 3:**
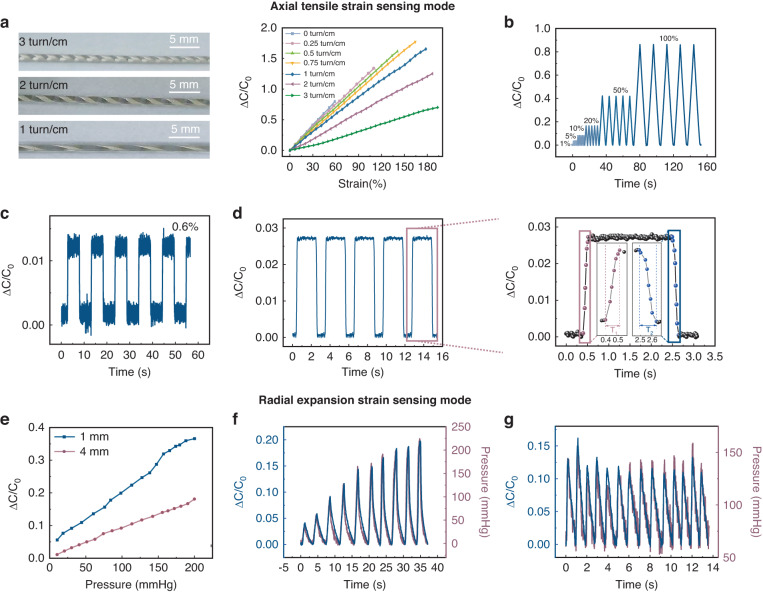
**Electrical output characteristics of dual-mode FSFCSS**. **a** Optical photography and capacitive response of FSFCSS with different helical turn densities. **b** Capacitive response of the FSFCSS under repeated 1%-100% axial tensile strain. **c** The detection limit of FSFCSS is 0.6%. **d** Response and recovery time of FSFCSS under 1% axial tensile strain at a stretching speed of 0.5 mm/s. **e** Capacitance response of FSFCSS with 1 mm and 4 mm inside diameters under radial expansion strain. **f** Pressure response curves measured by FSFCSS and a commercial pressure gauge under a continuous dynamic expansion pressure of 0–200 mmHg. **g** Capacitance and pressure response curves measured by FSFCSS and a commercial pressure gauge under 140 mmHg repeated pulse expansion pressure with 1 Hz

For the axial tensile strain sensing mode, based on the above theory, we further investigated the effect of turn density on the dual-mode sensing performance of FSFCSS. As shown in Fig. 3a, as the helical turn density increases from 0 to 3 turn/cm, the detection range of FSFCSS increases from 59 to 195%, while the sensitivity decreases from 1.347 to 0.381. The increased detection range can be attributed to the higher turn density of the helical Ag electrode, which allows for better dispersion and absorption of externally applied strain; as a result, the strain borne by each turn of the Ag electrode is reduced and the stretchability of the helical Ag electrode is enhanced^25–28^. Additionally, the higher turn density of the Ag electrode indicates that the relative displacement between each turn is smaller for a specific strain, resulting in a decrease in the sensitivity of the SFSCSS.

To balance the sensitivity and detection range, we prefer FSFCSS with a helical turn density of 1 turn/cm to be the optimal choice for subsequent characterization. As shown in Fig. 3b, c, FSFCSS exhibits stable and distinguishable responses to repeated tensile strains from 1% to 100% and demonstrates a minimum detection limit of 0.6%. Under a tensile speed of 0.5 mm/s, the response time and recovery time are 117 ms and 156 ms, respectively (Fig. 3d), and the hysteresis coefficient at 100% strain is only 1.44% (Supplementary Fig. S4). Moreover, after a 1200 cyclic test with 30% strain, the capacitance response of FSFCSS shows no observable degradation (Supplementary Fig. S5).

For the radial expansion strain sensing mode, a syringe pump was used to inflate the FSFCSS, and a commercial pressure gauge was utilized to record the expansion pressure. The expansion strain behavior of FSFCSSs with different diameters of 1 mm and 4 mm is first studied. As shown in Fig. 3e, FSFCSSs exhibit commendable linearity and sensitivity of 0.00171 mmHg^−1^ and 0.00086 mmHg^−1^, respectively. FSFCSS with a 1 mm diameter exhibits higher sensitivity due to its easier-to-obtain large expansion strain related to its small internal surface area. For continuous dynamic pressure and repeated pulse pressure variations, the capacitance response curves of FSFCSS indicate high consistency with the pressure curve, as shown in Fig. 3f, g. It is worth noting that even after over 12,000 cycles of expansion strain, no significant signal attenuation is found (Supplementary Fig. S6), demonstrating the potential of FSFCSS for applications requiring long-term continuous sensing.

Therefore, dual-mode FSFCSS demonstrates excellent sensing characteristics, such as a wide detection range, high sensitivity, good stability, fast response, and versatile operating modes. FSFCSSs with these characteristics are highly suitable for wearable and implantable health monitoring applications, such as fiber-shaped wearable sensors, implantable artificial ligaments, and smart muscles.

### Applications of FSFCSS in wearable health monitoring

**Fig. 4:**
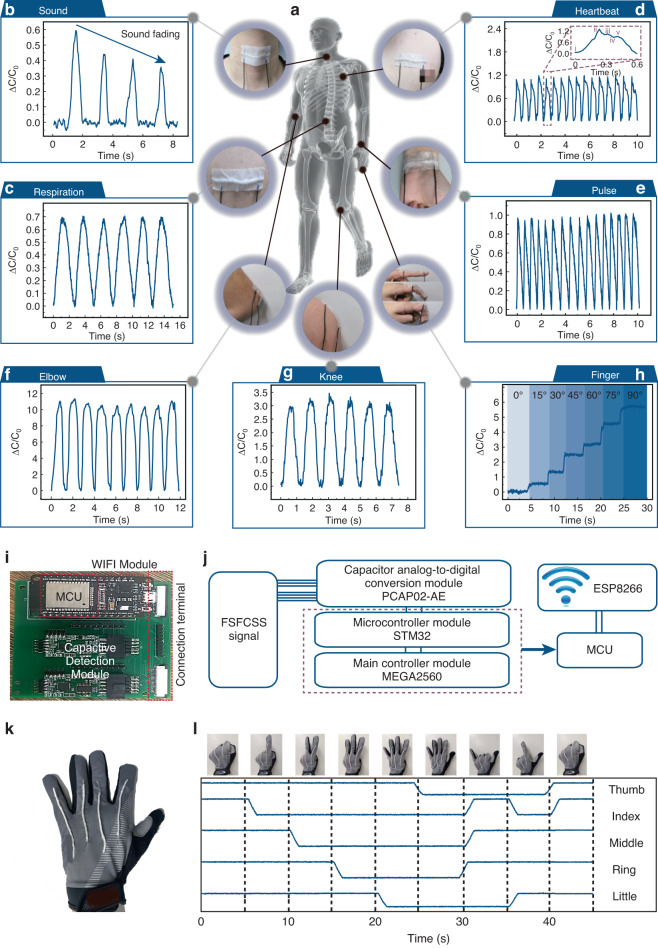
**Applications of FSFCSS in wearable health monitoring**. **a** Applications of FSFCSSs in wearable health monitoring, including **b** sound, **c** respiration, **d** heartbeat, **e** pulse, **f** elbow bending, **g** knee bending, and **h** finger bending. **i** Optical photograph and **j** schematic diagram of the portable data acquisition circuit board. **k**, **l** Applications of FSFCSS in human‒machine interactions for gesture recognition

Combining the excellent axial tensile strain sensing performance and fiber-shaped structure, the FSFCSS shows wide applications in several fields, including wearable health monitoring and human‒machine interaction. To evaluate the actual performance, we first affix our FSFCSS using 3M medical tape onto the skin surface of various parts of the human body, as shown in Fig. 4a, including the abdomen, chest, wrist, throat, knee, and fingers. Figure 4b–e shows the capacitance response curves of FSFCSS applied to different body parts and shows that FSFCSS can capture physiological signals such as sound, respiration, heart rate, and pulse in a clear and distinguishable manner. The measured physiological information of the volunteer is approximately a breath rate of 24 bpm, and heart rate and pulse display are identical at 96 bpm. Due to the ultrahigh sensitivity of FSFCSS, the recorded capacitance response curves contain rich physiological information. Taking the heartbeat signal as an example, the capacitive response curve recording captured all five typical feature points, including diastolic uprising, systolic peak point, systolic decline, valley, and the peak of the peripheral dicrotic notch^29,30^. In the era of big data, continuous real-time acquisition and analysis of human physiological health signals using FSFCSS contribute to the prevention and diagnosis of respiratory and cardiovascular diseases and the advancement of speech interaction applications. All these physiological data are collected noninvasively, rapidly, and in real time through user interaction, which will significantly impact health monitoring.

Moreover, the FSFCSS technology can be integrated into clothing and gloves, enabling real-time collection of human posture information and gesture movements. This capability opens up a wide range of applications in human‒machine interaction. As demonstrated in Fig. 4f–h, the capacitance variation curve of FSFCSS exhibits periodic changes during arm, knee, and finger bending. To capture multichannel capacitance signals of limb movements, a portable data acquisition circuit board was developed, as depicted in Fig. 4i, j. This circuit board includes a PCAP02-AE module for analog-to-digital conversion of capacitance signals, a microcontroller module STM32 for data preprocessing, a main controller module MEGA2560, and a WIFI module ESP8266 for data transmission. Furthermore, a smart glove application has been successfully demonstrated. By changing gestures in the order of “0”, “1”, “2”, “3”, “4”, “5”, “6”, “8”, and “0”, the corresponding high and low capacitance potentials can be distinguished and presented in a one-to-one correspondence, as shown in Fig. 4k, l. The gesture recognition function of FSFCSS can convert gestures into information, emotions, and commands, making it suitable for applications in human‒machine interaction, special effects production, VR interaction, and gesture-based operations.

### Applications of FSFCSS in hemodynamic monitoring

By utilizing the excellent radial expansion strain sensing characteristics of FSFCSS, FSFCSS could possibly be directly printed onto the surface of artificial blood vessels using DIW technology, enabling real-time acquisition of hemodynamic information. Coronary artery disease remains a leading cause of mortality worldwide^31^. Current treatment methods for severe coronary heart disease involve invasive coronary artery bypass surgery, which is aimed at improving blood flow in the heart vessels^32^. However, stent implantation poses potential risks, such as bleeding, allergies, vascular damage, and arteriovenous fistula, and leads to a higher risk of stent thrombosis and restenosis^33^. Therefore, real-time wireless monitoring of hemodynamic information is crucial for postoperative restenosis prevention.

**Fig. 5:**
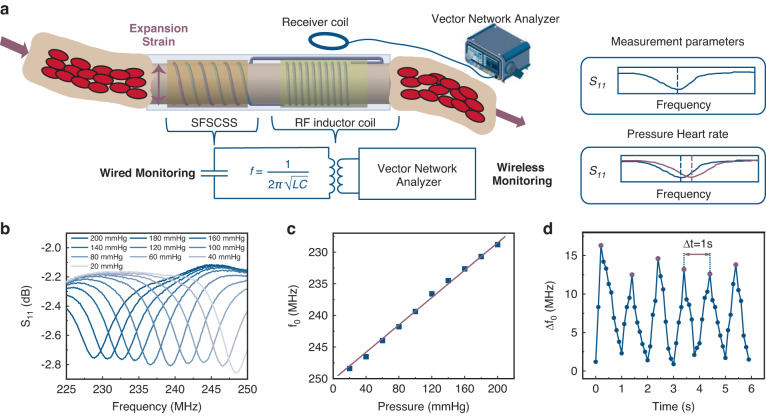
**Applications of FSFCSS in hemodynamic monitoring**. **a** Schematic diagram of the hemodynamic monitoring system. **b** The spectral signal of the hemodynamic sensor under 0–200 mmHg blood pressure. **c** The correlation between the resonance frequency of the wireless hemodynamic monitoring system corresponds to the stepwise blood pressure changes. **d** Measurement of the simulated 60 bpm heart rate using a hemodynamic monitoring system

To develop an implantable wireless hemodynamic sensor, we fabricated FSFCSS on the surface of TPU tubular fibers and processed a single helical silver electrode as an RF coil using the DIW technique. TPU is commonly used in the manufacturing of artificial blood vessels. A typical L-C circuit is formed by connecting FSFCSS in series with the printed RF coil. A schematic diagram of the hemodynamic sensing system is displayed in Fig. 5a. During the measurement process, the pulsatile changes in blood pressure induce radial expansion strain in FSFCSS, resulting in periodic changes in capacitance. This capacitance variation is converted into a frequency shift phenomenon in the resonant frequency (*f*_*0*_) of the L-C circuit through the RF inductor coil. We achieved wireless measurement by employing a mature RF coupling method based on a vector network analyzer.

To evaluate the performance of this wireless hemodynamic sensing system, we connected a programmable syringe pump to the TPU tubular fiber to simulate blood pressure changes. Initially, we tested the wireless transmission capability of the printed RF coil. Through experiments, we observed that even at a wireless transmission distance of 3 cm, the hemodynamic sensing system was able to obtain clear spectral signals (Supplementary Fig. S7). The interlayers between the transmitting and receiving coils can cause attenuation of the RF signal of the vector network analyzer, resulting in distortion of the results. Research shows that simulated interlayers have minimal impact on wireless signal transmission, including palms (1.5 cm thickness), Ecoflex film (1.5 cm thickness), and immersion in phosphate-buffered saline with 1.5 cm thickness, demonstrating the system’s ability to wirelessly monitor hemodynamic information under implantation conditions (Supplementary Fig. S8). Subsequently, we used the hemodynamic sensor to measure simulated changes in blood pressure and heart rate. The experimental results revealed significant frequency shifts in the spectral signals of the hemodynamic sensor within a blood pressure range of 0 to 200 mmHg (Fig. 5b, c). As blood pressure increased, the system’s resonance frequency decreased from 248.39 MHz to 228.89 MHz, with a resolution of 2.16 MHz/20 mmHg. Additionally, we simulated pulse pressure changes at a heart rate of 60 bpm, and the results showed that the peak frequency *Δf*_*0*_ extracted from the continuous spectral signal was 1 Hz, consistent with the preset heart rate of 60 bpm. These results demonstrated the ability of the device to wirelessly monitor arterial pulse waves (Fig. 5d).

The FSFCSS, customized and fabricated based on DIW technology, exhibits excellent dual-mode sensing characteristics and electromechanical performance, combining a wide detection range with high sensitivity. FSFCSS could capture subtle skin vibrations and joint strain, enabling real-time and continuous capture of physiological health signals and gesture signals, thereby contributing to the advancement in wearable health monitoring and human‒machine interaction. Additionally, FSFCSS conceptually validates the wireless monitoring system for hemodynamics, providing new insights for the development of smart integrated sensors directly integrated on portable vascular surfaces. However, the stability in complex in vivo environments and reliability in long-term usage of FSFCSS need further verification; the substrate material needs to be replaced with transplantable artificial blood vessels; and whether the printing process affects the performance of artificial blood vessels should be investigated.

## Conclusions

In this study, we successfully fabricated a dual-mode FSFCSS on a TPU fiber substrate using a high-precision DIW printing device. We comprehensively analyze the working mechanisms of the sensor in axial tensile strain sensing and radial expansion strain sensing modes. For the axial tensile strain sensing mode, FSFCSS exhibits excellent performance, including a wide detection range (178%), significant sensitivity (0.924), low detection limit (0.6%), fast response and recovery times (117 and 156 ms), low hysteresis coefficient (1.44%), and excellent mechanical stability. For the radial expansion strain sensing mode, the FSFCSS demonstrates an outstanding response to dynamic and pulse pressure changes, with a high sensitivity of 0.00086 mmHg^−1^. Therefore, we explored the application of FSFCSS in the field of wearable health monitoring through several perspectives, including monitoring human physiological signals, real-time collection of human posture information, and human‒machine interactive applications. Furthermore, we developed a wireless hemodynamic sensor by connecting FSFCSS with a printed single helical RF coil, demonstrating the feasibility of this sensor for implantable hemodynamic monitoring. This work not only introduces a new method for fabricating fiber-shaped capacitive strain sensors but also contributes to the research and development of wearable and implantable health monitoring.

## Materials and methods

### Chemicals and materials

The thermoplastic polyurethane (TPU) fiber substrate (diameter: 500–2000 μm) was purchased from Shengyi Plastic Insulation Materials Co., Ltd., China. The polyurethane tube substrate (inner/outer diameter 600/1000 μm, 3600/4000 μm) was purchased from Shanghai Zhecheng Plastic Products Co., Ltd., China. Ag ink (BASE-SC01, 15000–40000 cps) was purchased from Shanghai Mifang Electronic Technology Co., Ltd. BaTiO_3_ (BTO) nanoparticles were obtained from Heowns Opde Technology Co., Ltd., Tianjin. Ecoflex (00–30) was purchased from Smooth-On Inc., Macungie, PA, USA. All of the chemicals were directly used as received without further purification.

### Fabrication of FSFCSS

(1) A cleaning agent and deionized water were used to manually remove the protective grease on the surface of TPU fibers. The TPU fibers were ultrasonicated in anhydrous ethanol at a power of 180 W for 10 min and dried with nitrogen gas flow for use. (2) Activate TPU fibers by PLASMA with an oxygen flow at a pressure of 17 Pa and a power of 300 W for 30 s. (3) Print parallel helical-structured Ag ink electrodes on the TPU fibers by self-built DIW printing equipment with different parameters. At the same time, a heating platform with a distance of 1 cm was used to quickly dry the Ag ink at 80 °C, and then the sample was further dried in an 80 °C oven for 20 min. (4) The BTO powder was mixed with Ecoflex with a 10 wt% weight ratio, and the BTO@Ecoflex mixture was applied evenly on the printed TPU fiber surface. Then, the samples were fixed on the synchronous motors by JTO fixtures and rotated at a speed of 60 rpm until the mixture was completely cured.

### Fabrication of a hemodynamic sensor based on FSFCSS

(1) Fabrication of an FSFCSS on the surface of a PU fiber. (2) Print a single helical Ag electrode beside the FSFCSS as the RF coil and package it with PU solution. (3) Connect FSFCSS and a single helical RF coil in series by DIW printing equipment and encapsulate them with Ecoflex. (4) The wireless hemodynamic sensor based on a typical L-C resonant circuit is successfully manufactured.

### Characterization of FSFCSS

The surface and cross-section morphologies of the FSFCSSs were examined using a field emission scanning electron microscope (FEI, Nova Nanosem 430). The electrode linewidth and thickness were measured by a profilometer (Alpha-Step IQ). The tensile properties of the TPU fibers were tested by an electric tensile testing machine (Dongguan Zhiqu Precision Instrument, ZQ-990B) with a tensile rate of 0.834 mm/s.

### FSFCSS for axial tensile strain sensing

(1) For axial tensile strain sensing, a linear motor (LinMot 1100) was used to provide tensile strain to FSFCSS, and an LCR impedance analyzer (Agilent, E4980A) was used to measure the capacitance response of FSFCSS. (2) For physiological signal monitoring, 3M medical tape was utilized to securely attach FSFCSS to human skin, and an LCR impedance analyzer was used to measure the capacitance response. (3) For human‒machine interactive applications, five FSFCSSs were sewn into a commercial glove and combined with a portable signal processing circuit to constitute a smart glove. The signal processing circuit contains an analog-to-digital converter (ADC) module (PCAP02-AE), a microcontroller module (STM32), the main controller module (MEGA2560), and a WIFI module (ESP8266).

### FSFCSS for radial expansion strain sensing and hemodynamic monitoring

(1) For radial expansion strain sensing, a syringe pump (KDS-210) provides static expansion strain, dynamic expansion strain, and pulse expansion strain, and an LCR impedance analyzer and commercial pressure sensor (Honeywell, 26PCBFB6G) were used to synchronously measure the capacitance response of the FSFCSS and expansion pressure. (2) For hemodynamic monitoring, the syringe pump was used to simulate blood pressure changes of 0–200 mmHg with a heartbeat of 60 bpm, and the Vector Network Analyzer was used to wirelessly measure hemodynamic information.

### Supplementary information

supplemental material
