# Supplementary material for: A dual-mode fiber-shaped flexible capacitive strain sensor fabricated by direct ink writing technology for wearable and implantable health monitoring applications
Source: Microsyst Nanoeng. 2023 Dec 21;9:158. doi: 10.1038/s41378-023-00634-9 (PMC10739884; doi:10.1038/s41378-023-00634-9)
Supplement: Supplementary file 1 — supplemental material [file 41378_2023_634_MOESM1_ESM.docx]

Supporting information

A Dual-Mode Fiber-Shaped Flexible Capacitive Strain Sensor Fabricated By Direct-Ink-Writing Technology For Wearable And Implantable Health Monitoring Applications

Chi Zhang^+^, Wenyu Ouyang^+^, Lei Zhang ^*^, Dachao Li^*^

^+^C. Zhang and W. Ouyang contributed equally to this work.

C. Zhang, W. Ouyang, L. Zhang, D. Li

State Key Laboratory of Precision Measuring Technology and Instruments, Tianjin University, Tianjin, 300072, China.

E-mail: zhangleitd@tju.edu.cn; dchli@tju.edu.cn

**Content of the Supplementary information**

Fig. S1: Cross-sectional SEM image of the printed helical Ag electrode.

Fig. S2: Mechanical properties of single TPU fiber.

Fig. S3: The tensile conductivity of helical Ag electrodes.

Fig. S4: The hysteresis coefficient of FSFCSS.

Fig. S5: Stability of the FSFCSS under repeatable axial tensile strain.

Fig. S6: Stability of the FSFCSS under repeatable radial expansion strain.

Fig. S7: The spectrum signals with different wireless transmission distances.

Fig. S8: The spectrum signals with a different interlayer between the transmitting and receiving coil.


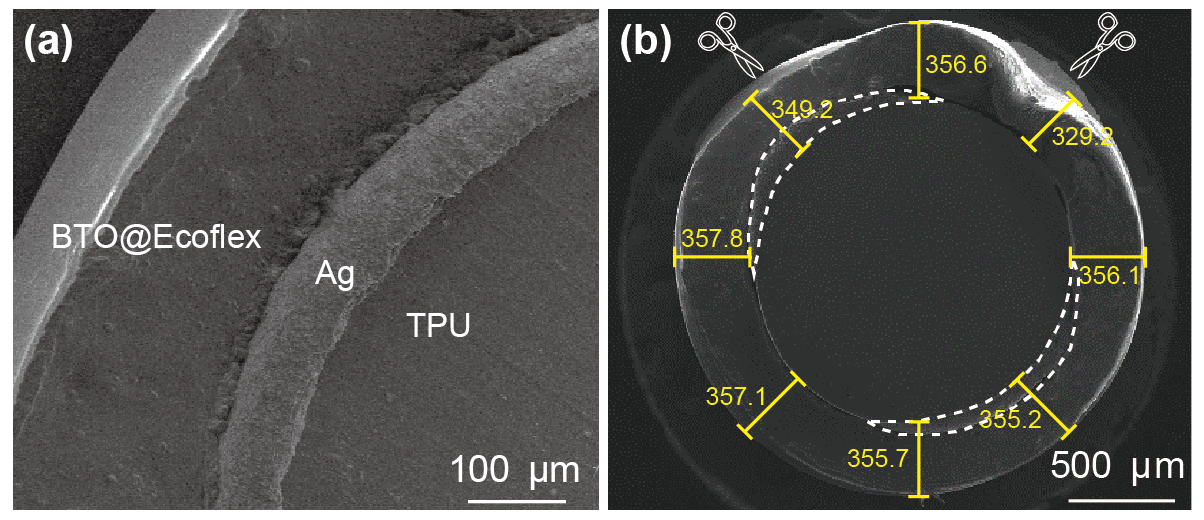


**Fig. S1** (a) The cross-sectional SEM image of the interface between printed Ag electrode and TPU fiber. (b) The cross-sectional SEM image of Ag/TPU/BTO@Ecoflex sample with 8 thickness scales.


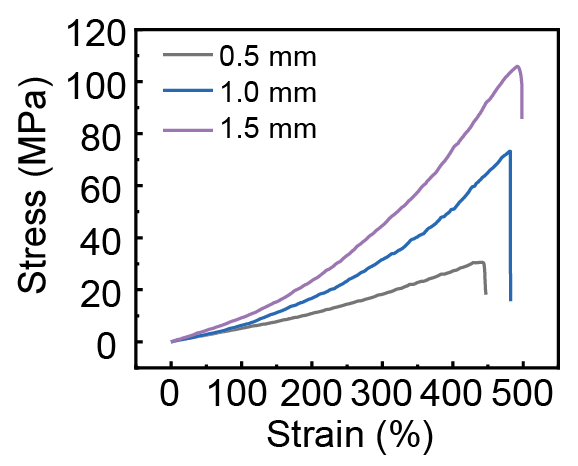


**Fig. S2** Stress-strain curves of pure TPU fibers with diameters of 0.5, 1.0 and 1.5 mm.


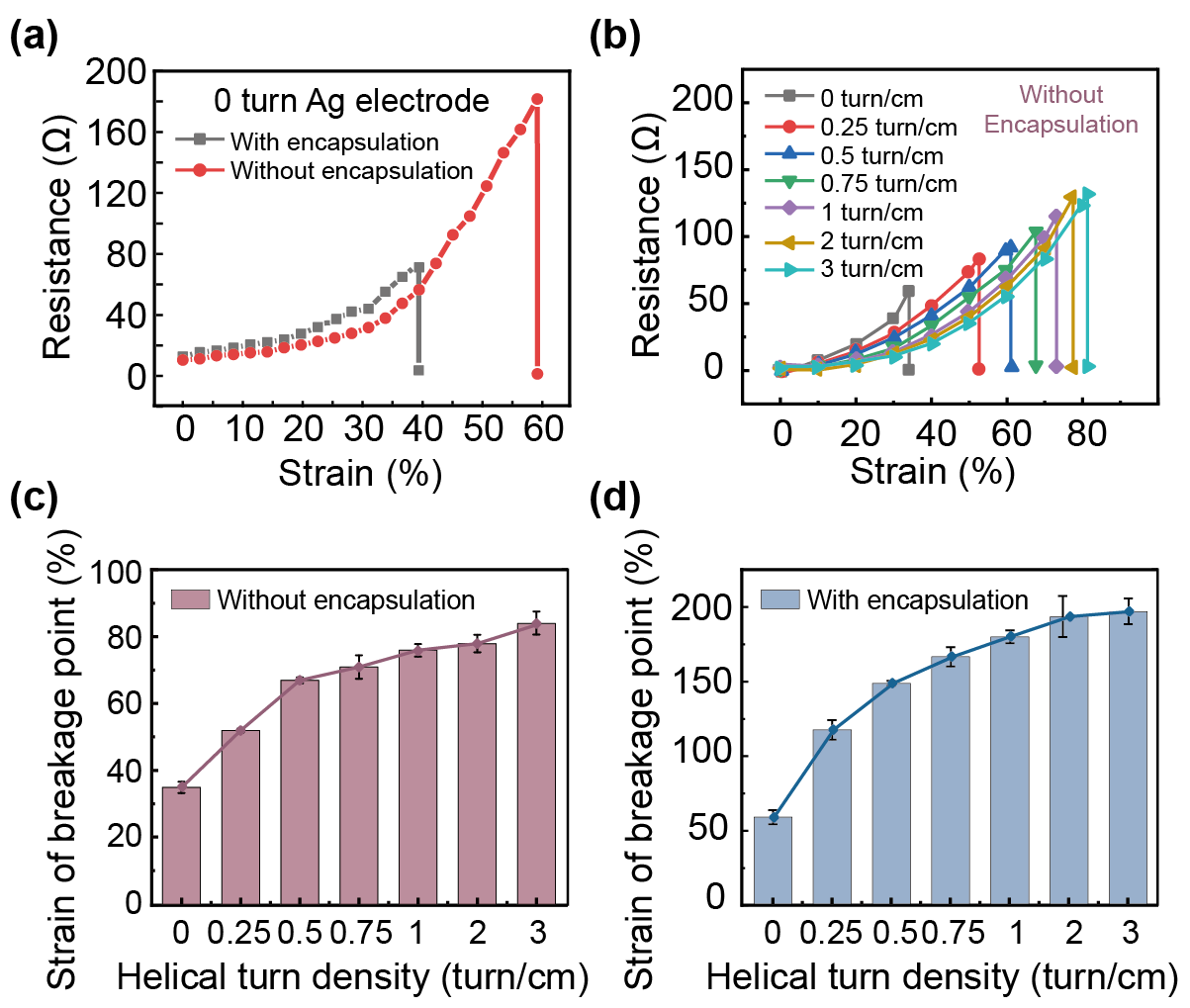


**Fig. S3 (a)** Conductivity of TPU fiber printed linear Ag electrodes (0 turns/cm) before and after encapsulating. (b) Resistance-strain curves of Ag electrodes with different turn densities without encapsulation. Comparison of strain of breakage point with different printed Ag electrode (c) without and (d) with encapsulating by the BTO@Ecoflex..


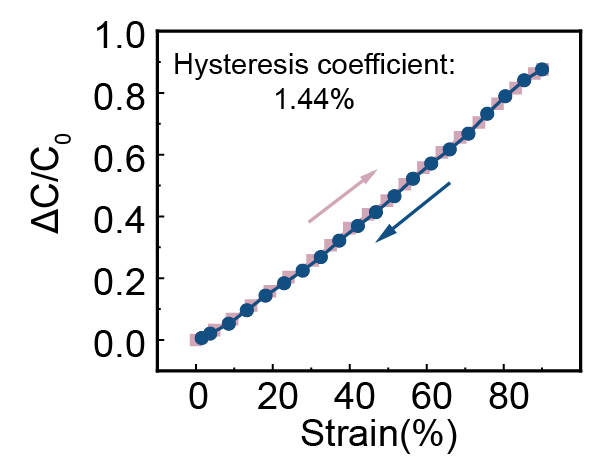


**Fig. S4** The relative capacitance changes and hysteresis coefficient of FSFCSS under axial 90% strain.


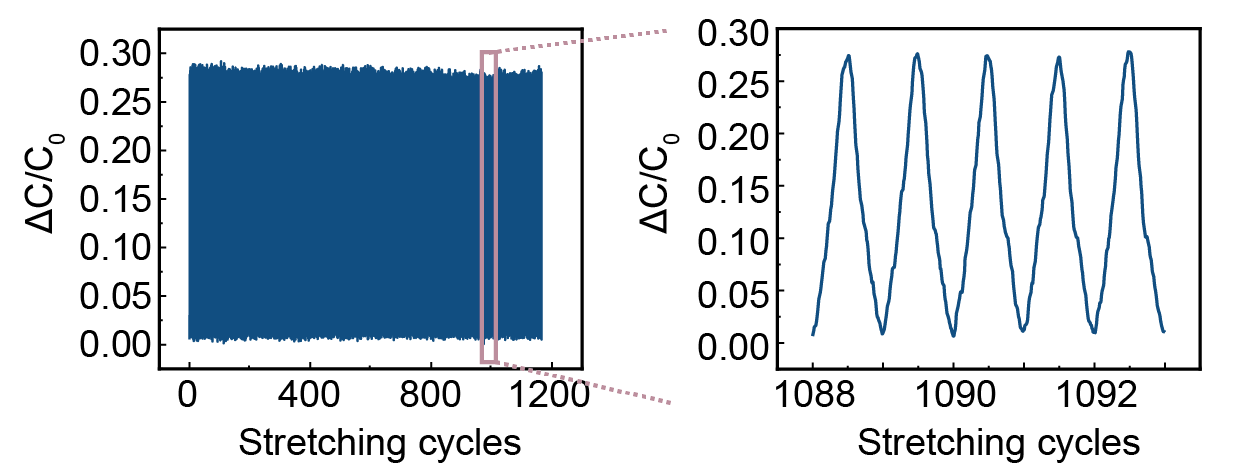


**Fig. S5** Stability of the FSFCSS under repeated 30% axial tensile strain over 1,200 cycles.


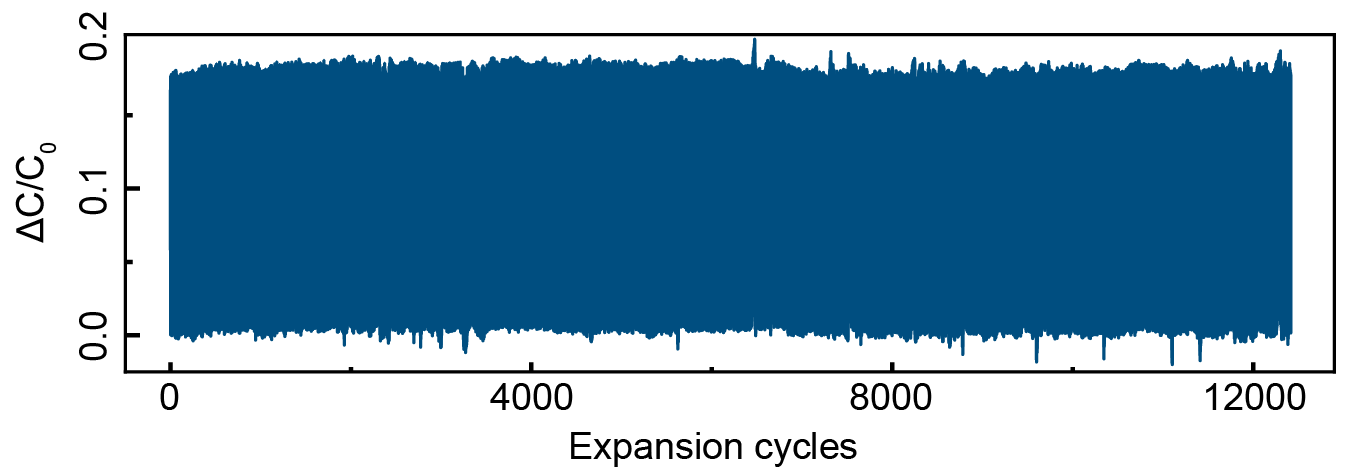


**Fig. S6** Stability of the FSFCSS under radial expansion strain over 1,2000 cycles.


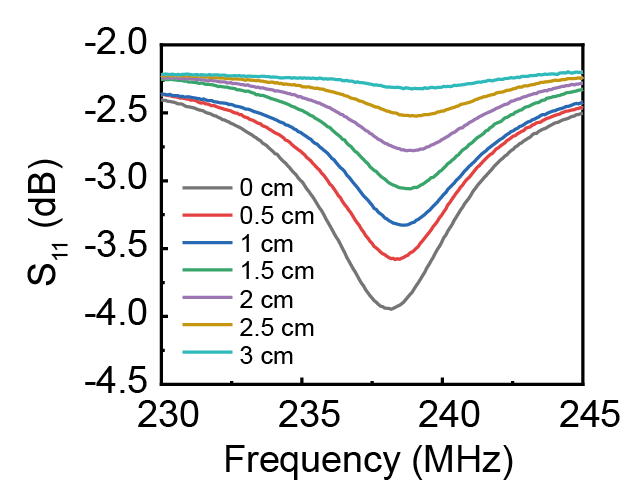


**Fig. S7** The influence of wireless transmission distance on the performance of hemodynamic sensors.


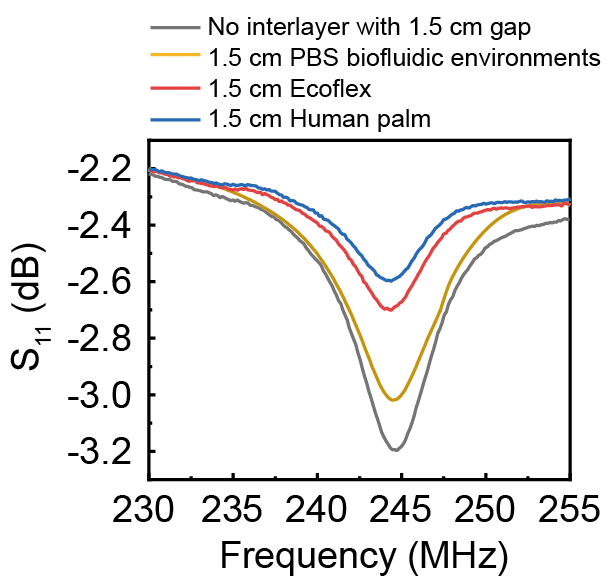


**Fig. S8** The influence of the interlayer between transmitting and receiving coil on the performance of hemodynamic sensors.
